# Residents or Consultants: Who Writes Orders for Patients Taken Care of by the Internal Medicine Residency Service?

**DOI:** 10.7759/cureus.6653

**Published:** 2020-01-14

**Authors:** Parmatma Parajuli, Saeed Ali, Shubhechchha Singh, Jian Guan, Manoucher Manoucheri

**Affiliations:** 1 Internal Medicine, AdventHealth Orlando, Orlando, USA; 2 Medicine, Florida Hospital, Orlando, USA; 3 Internal Medicine, Florida Hospital, Orlando, USA

**Keywords:** residents, specialists, consultants, orders

## Abstract

Background and aims

The Accreditation Council for Graduate Medical Education (ACGME) mandates that Internal Medicine residents shall place all the orders for their patients. The purpose of this rule is to assure comprehensive knowledge of patient information and direct involvement in decision-making. However, there is a general perception that a large proportion of orders for patients taken care of by the residents are being written by consultants or other providers. The objective of the study was to determine the proportion of routine orders placed by Internal Medicine residents in comparison to consultants/subspecialty providers for patients under the care of the Internal Medicine Residency Service (IMRS).

Material and methods

All the orders on patients admitted to the IMRS at AdventHealth Orlando from July 9, 2017, to July 15, 2017, were documented. Of these, Emergency Department (ED) orders, “STAT/ASAP/NOW orders,” “discharge by consultant” orders, and “consent for procedure” orders were excluded. The main outcome measure was the proportion of orders placed by Internal Medicine residents as compared to consultants and all other providers.

Results

A total of 6471 orders placed on 90 patients admitted to the IMRS and with at least one consultant were included in the study. Of them, 96.8% of all orders were placed by Internal Medicine residents. Only 3.1% of all orders were placed by consultants and other providers. Of them, the majority of the orders were specialty-specific orders and were appropriate. Only 1.1% of all orders were “routine” orders placed inappropriately by consultants and other providers. A total of 121 consultations were made, and there were no new consultations initiated by consultants and other providers during the study period.

Conclusion

The vast majority of orders for patients taken care of by the IMRS were placed by the Internal Medicine residents themselves. Only a very small proportion of the orders were placed by consultants and other providers in this limited timeframe study. The findings are consistent with the ACGME mandate that residents write all orders for patients under their care except in special circumstances.

## Introduction

The use of the electronic health record (EHR) has become increasingly common in US medical institutions [1]. In teaching hospitals where an EHR is available, residents are encouraged to place orders, retrieve data, enter data, and perform clinical documentation on inpatients and outpatients. Several education-based studies have been conducted to evaluate the impact of EHR on the training of medical students and residents and its potential as an educational tool [2-4].

The Accreditation Council for Graduate Medical Education (ACGME) mandates that residents should write all routine orders for patients admitted under their care, with appropriate supervision by the attending physician [5]. This ensures resident autonomy and involvement in the care of the patient. The ACGME primarily relies upon the annual resident survey to determine the level of compliance with this rule. We are not aware of any previous study that has determined the actual level of compliance in the Internal Medicine residency programs and hospitals. However, it has been a common perception that significant numbers of routine orders and new consult requests are placed by consultants and non-internal medicine residents.

The objectives of this study are: (1) to determine the percentage of routine orders placed by Internal Medicine residents for patients under their care and (2) to determine the proportion and appropriateness of orders written by consultants or other providers.

## Materials and methods

This is a retrospective quality improvement project conducted in Florida Hospital Orlando (currently AdventHealth Orlando) from July 9, 2017, to July 15, 2017. All 90 patients who were under the care of IMRS during this time period were enrolled in the study. A total of 6542 orders were placed on these patients. After the exclusion of departmental orders, “discharge by consultant” orders, and “consent for a procedure” orders, 6471 orders were included in the study. The study was approved by the Institutional Review Board and the Ethics Committee of the hospital.

Appropriate orders (by consultants or other providers) were defined as specialty-specific orders, such as dialysis orders, anesthesia orders, pre-surgical orders, surgical orders, and post-surgical orders.

Inappropriate orders (by consultants or other providers, including Physician Assistants or Nurse Practitioners) were defined as routine orders, which should have been put as a recommendation in their notes and could have been placed by residents.

## Results

**Figure 1:**
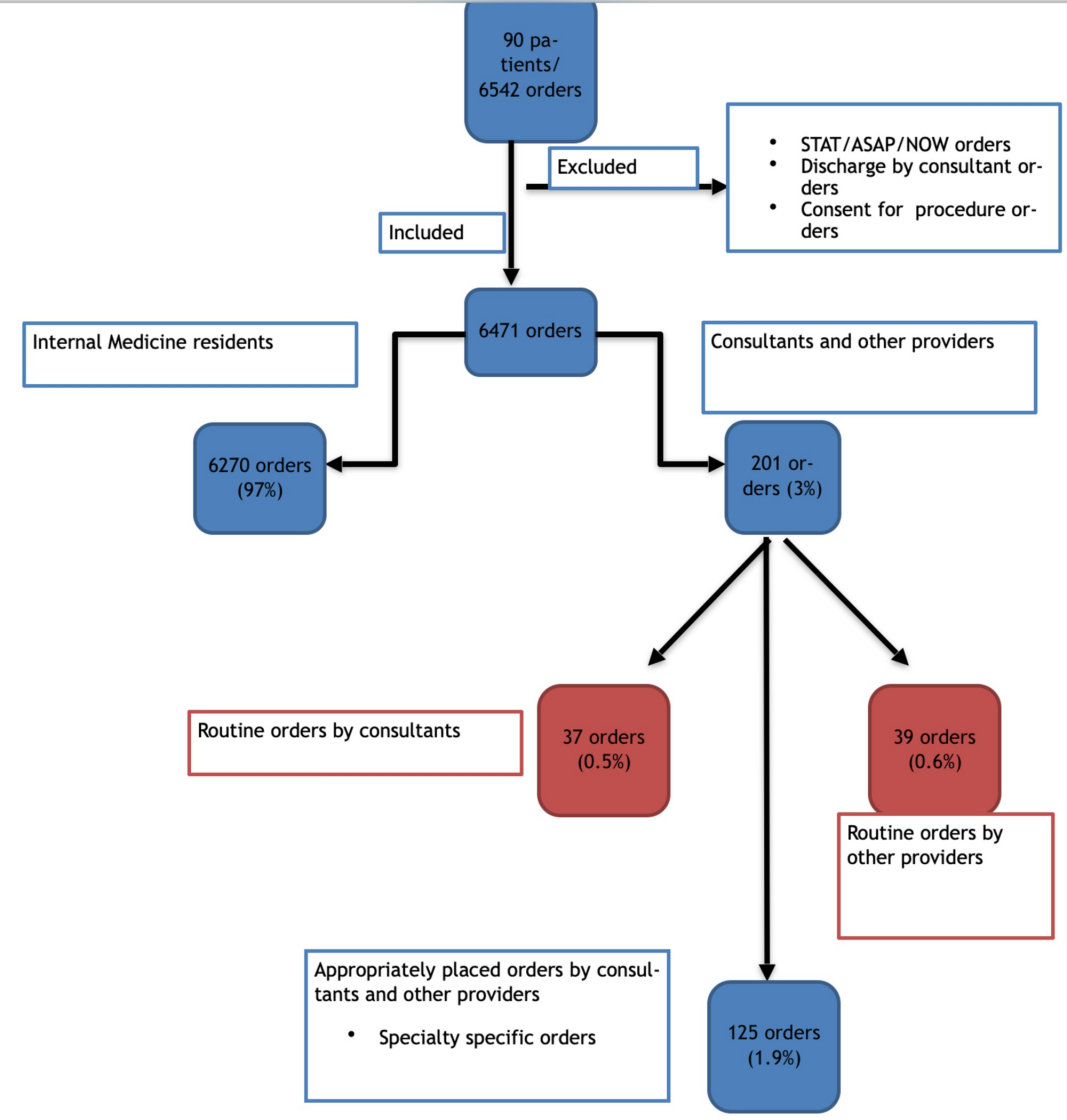
Flow chart summarizing the findings. Blue color depicts appropriately placed orders, Red depicts inappropriately placed orders.

Of the 6471 orders included in the study, 96.8% (n=6270) of all orders were placed by the Internal Medicine residents. Only 3.1% of all orders (n=201) were placed by consultants and other providers. Of them, the total number of routine orders inappropriately placed by consultants was 37 (0.54% of all orders) and the total number of routine orders placed by other providers was 39 (0.57% of all orders), making it a total of 76 orders (1.1%). The remaining 125 orders placed by consultants and other providers were specialty-specific, appropriately placed orders (Figure 1). A total of 121 consultation requests were placed on the 90 studied patients. There were no new consultation requests placed by the consultants during the period studied.

## Discussion

Graduate medical education develops physicians who focus on excellence in the delivery of safe, equitable, affordable, and quality care, and on the health of the populations they serve. The use of EHR and the practice of writing appropriate orders under supervision is an integral part of medical education and the delivery of safe and quality care. There have been several studies that have evaluated the usage of EHR in academic health institutions in the United States, the impact of EHR on the training of medical students/residents, and its value as an educational tool [1-4]. However, there is a paucity of studies looking into the proportion and appropriateness of orders written by Internal Medicine residents in comparison to that by consultants/specialists.

The ACGME mandates that residents must write all orders for patients under their care, with appropriate supervision by the attending physician. In unusual circumstances when an attending physician or subspecialty physician writes an order on a resident's patient, the attending or subspecialty physician must communicate his or her action to the resident in a timely manner [5]. We designed this study to quantify the proportion of orders written by Internal Medicine residents for patients under their care in comparison to that by consultants/specialists from other services in the same patients. We also assessed the appropriateness of the orders written by consultants or subspecialty providers.

Our study demonstrates that a vast majority of the orders for patients admitted to the IMRS are placed by the Internal Medicine residents. The proportions of orders placed on the same subset of patients by consultants and other providers were very small and only 1.1% of all orders were placed inappropriately. The majority of the orders placed by consultants and other providers were appropriately placed specialty-specific orders, which were in the best interests of the patients. We also did not find new consultations placed by consultants and other providers over the study time frame. Overall, the practice of placement of orders for patients admitted to the IMRS by the Internal Medicine residents themselves is consistent with ACGME rules.

This is a remarkable finding in the context of an academic program working in conjunction with a large number of private sub-specialty services in a tertiary-level community hospital with a large patient burden in need of specialized care. The findings are in contrast to the general perception that consultants place the majority of orders instead of Internal Medicine residents. This may be a reflection of the efforts by the residents to establish a direct line of communication between the services, before or as soon as the consult requests are placed. In addition, the hospital EHR software notifications directed to consultants requesting them to communicate with the primary service before orders are put in may also have contributed to the findings. Overall, these efforts may contribute to high-value care while minimizing the duplication of orders and, at the same time, help maintain mutual respect between services. Most importantly, they would strengthen a sense of ownership for the residents, which is fundamental to their training.

There are some major limitations to this study. This is a single-center study conducted in orders placed over a very short time period. Besides, this study does not take into account the variability amongst different specialty services. A larger study conducted over a longer time frame, with more sub-specialty specific analysis, may be needed to further assess the extent of this perceived problem.

## Conclusions

In this study, it was demonstrated that the majority of the orders for patients taken care of by the Internal Medicine Residency Service were placed by the residents themselves despite working in conjunction with a large number of specialty services in a large tertiary-level center. The findings highlight the importance of efforts to establish prompt communication between residents and specialists, which may contribute to high-value medical care and education.
